# Potential Ascertainment Bias and Event Adjudication in T Cell Engager–Associated Cardiovascular Event Reporting

**DOI:** 10.1016/j.jaccao.2026.04.011

**Published:** 2026-08-25

**Authors:** Syed Muhammad Ali, Valle Chiang, Mahmood Ahmad

Itzhaki Ben Zadok et al^1^ provided an important systematic characterization of cardiovascular events (CVEs) during bispecific T cell engager therapy, with rigorous competing-risk methods and clearly prespecified endpoint definitions. We wish to raise 2 methodological considerations that could affect interpretation of the incidence estimates and the association between grade ≥2 cytokine-release syndrome (CRS) or immune effector cell–associated neurotoxicity syndrome (ICANS) and CVE risk.

Concerning differential surveillance intensity, the investigators acknowledged that cardiac testing was not prespecified and that biomarker acquisition was inconsistent throughout the defined study period.^1^ We believe that this has the potential to lead to a meaningful risk for ascertainment bias in the key exposure-outcome association. Patients developing grade ≥2 CRS and/or ICANS are, by and large, clinically “sicker” and more closely monitored. These patients are also more likely to undergo electrocardiography, telemetry, troponin assessment, and echocardiography, increasing the probability that arrhythmias or left ventricular dysfunction are identified and documented. Such differential outcome detection is a recognized source of bias in unblinded and observational studies.^2^^,^^3^ We would respectfully encourage the investigators to report, even descriptively, the proportion of patients that underwent on-treatment echocardiography and the frequency of troponin measurement stratified by CRS or ICANS severity. A sensitivity analysis restricted to clinically overt, hospitalization-level events, such as acute myocardial infarction, stroke and heart failure hospitalization, might help determine whether the observed associations persist.

Beyond surveillance intensity, the process of event adjudication warrants further consideration. Several CVE components, such as heart failure with preserved ejection fraction, confirmed by diuretic response and arrhythmias requiring intravenous rate control, are sensitive to documenting practices and clinical interpretation.^1^ We seek clarity to whether these events were confirmed by a reviewer independent of the treating team or were reconciled across dual reviewers. We believe that this would strengthen the confidence in the ascertainment process and assist readers seeking to replicate these criteria prospectively.

We do not believe these points diminish the significance of this contribution, which address an important gap in cardio-oncology literature. However, we believe that addressing these concerns would substantially advance the evidence base for risk stratification in this expanding therapeutic area.
